# Consumer Acceptance of Dry Dog Food Variations

**DOI:** 10.3390/ani4020313

**Published:** 2014-06-16

**Authors:** Brizio Di Donfrancesco, Kadri Koppel, Marianne Swaney-Stueve, Edgar Chambers

**Affiliations:** The Sensory Analysis Center, Department of Human Nutrition, Ice Hall, Kansas State University, Manhattan, KS 66506, USA; E-Mails: briziod@ksu.edu (B.D.); Marianness@ksu.edu (M.S.-S.); eciv@ksu.edu (E.C.)

**Keywords:** appearance, aroma, consumer, dog, liking, pet food

## Abstract

**Simple Summary:**

The objectives of this study were to compare the acceptance of different dry dog food products by consumers, determine consumer clusters for acceptance, and identify the characteristics of dog food that drive consumer acceptance. Pet owners evaluated dry dog food samples available in the US market. The results indicated that appearance of the sample, especially the color, influenced pet owner’s overall liking more than the aroma of the product.

**Abstract:**

The objectives of this study were to compare the acceptance of different dry dog food products by consumers, determine consumer clusters for acceptance, and identify the characteristics of dog food that drive consumer acceptance. Eight dry dog food samples available in the US market were evaluated by pet owners. In this study, consumers evaluated overall liking, aroma, and appearance liking of the products. Consumers were also asked to predict their purchase intent, their dog’s liking, and cost of the samples. The results indicated that appearance of the sample, especially the color, influenced pet owner’s overall liking more than the aroma of the product. Overall liking clusters were not related to income, age, gender, or education, indicating that general consumer demographics do not appear to play a main role in individual consumer acceptance of dog food products.

## 1. Introduction

Pet food buyers, despite economic recession challenges and subsequent money-saving trends, seem to be more than willing to spend money on pet food. A recent survey [1] indicated nearly one-third of consumers “preferred to shop at pet product retailers that offer the best products available, even if they are more expensive”. Another survey on pet owners showed that 21% of US dog owners spend an average of US $100 or more per month on their dogs [2]. The pet food industry benefits from this consumer behavior and is focusing even more on premium products. In the pet food market, this means natural ingredients and a focus on health issues.

Factors such as brand and package contribute to the formation of expectation, the selection process, and the purchase intention of a product by consumers [3]. For those products that can be experienced only after purchase and in categories where multiple products are available for the consumer, such as the pet food category, brand represents a signal of quality and can help in the selection process [4]. Moreover, considering that pet food will not be directly consumed by owners, and therefore complete feedback cannot be provided, branding can hold a much higher importance. Packaging in the pet food industry conveys information on the particular properties of ‘premium’ products or natural and healthy ingredients, increasing with humanization of pets by owners. When brand and packaging information are removed, only sensory properties such as appearance and aroma contribute to the consumer’s acceptance of the pet food, with the result being important especially for the repurchase of a product by owners [5].

Human sensory analysis with pet foods has not been frequent. A few studies have been conducted with pet food [6] and cat foods [7,8]. Di Donfrancesco *et al*. [9] looked at a variety of dry dog food products are available in the US market with varying appearance, aroma, flavor, and texture sensory properties, demonstrated by recent studies. These authors showed that some common aroma attributes, such as barnyard, grain, brothy, oxidized oil, cardboard, and stale are common sensory characteristics in this category. Other aroma notes such as meaty, liver, or fishy are more product-specific. Dry dog food products also were shown to be different for appearance characteristics such as surface roughness, oily appearance, porousness, and fibrousness.

In the United States and other developed countries most pet dogs and cats are fed commercial food, with about 50% of dogs fed entirely with dry commercial food [10]. Those authors noted that the most common is to feed dogs two times per day and provide one or more treats each day. Laflamme *et al*. [10] also observed that 31% of owners watch their dogs eat. This information suggests a high daily exposure of dog owners to pet food. Pet owners interact with pet food when feeding their pet and because of the lack of linguistic capability of pets, they can often determine pet food acceptability, in the same way parents can do with infants [11]. Understanding the boundaries that industry can push during product development without having a negative impact on pet owners’ acceptability can be fundamental.

Sensory analysis techniques, both analytical and hedonic, can help in this process to determine properties such as appearance, texture, aroma and flavor that are important for pet food acceptability. It has been shown that ‘taste plus flavor’ is considered the most important factor for consumers when experiencing food and beverages [12]. Other sensory characteristics such as aroma and appearance can assume a higher relevance when trying to assess humans’ pet food acceptability, since humans wouldn’t typically taste pet food.

The objectives of this study were to: (1) understand consumers’ acceptance of various different commercial dry dog food products without branding or packaging; (2) identify consumer clusters and determine if these clusters relate to age, income, gender, or education; and (3) isolate drivers of consumers’ liking and purchase intent.

## 2. Materials and Methods

### 2.1. Samples

Eight samples were purchased from local pet stores, grocery stores, and discount stores. Samples were different in brand, type of kibble, price, and presence of specialty ingredients (Table 1). Sample A (3 kibbles) and sample C (4 kibbles) were each comprised of multiple kibbles, differing in size, shape and color. The samples were chosen based on their aroma and appearance characteristics measured by Di Donfrancesco *et al*. [9]. In that study, samples were small (D, G), medium (A, B, E, F, H), and large (one kibble type of sample A). Some samples were nugget-shaped (A, B, E, G, H), other were oval-shaped (kibble in sample A), ‘o-shaped’ (D), square-shaped (F), or they showed a miscellaneous shape such as one kibble in sample A. The choice of some of the samples was based on preliminary descriptive data collection in order to have a wide range of aroma profiles within the sample set. Some samples had higher fishy aroma notes (G, H) while others, such as sample E, had higher grain aroma notes. Sample D had high oxidized aroma levels and sample A showed plastic aroma notes. Some samples (F and D) presented higher meaty aroma notes than the others and sample G showed higher levels of liver aroma.

All samples were evaluated within the “best by” date on the package and all sample lots were checked to ensure they had not been subject to a product recall. The products were purchased one week before testing and were stored at room temperature.

### 2.2. Descriptive Sensory Analysis

Six highly trained panelists from the Sensory Analysis Center, Kansas State University (Manhattan, KS, USA) analyzed the samples for aroma and appearance attributes. Each of the panelists had more than 120 hours of descriptive analysis panel training which included techniques and practice in attribute identification, terminology development, and intensity scoring. Moreover, each assessor had more than 1,000 hours of experience with a variety of food products, including dried dog food, and had conducted studies using the consensus method (used in this study).

Attribute intensities were evaluated on a scale where 0 = none and 15 = very high. Samples were first evaluated individually by panelists, then the final aroma and appearance profile was developed after a discussion led by the panel leader to determine the consensus score for each attribute. Similar procedures have been used recently in other sensory studies [13,14,15]. The lexicon used in descriptive profiling was developed and used by Di Donfrancesco *et al.* [9].

Each sample was prepared 30 minutes prior to testing and was served in a ~100 mL plastic cup for appearance evaluation. For aroma evaluation, 3 grams of sample was weighed in a medium glass snifter, and covered with a watch glass. Samples were coded with three-digit random numbers. The testing room was maintained at 21 ± 1 °C and 55 ± 5% relative humidity. 

**Table 1:** Dry dog food samples and ingredients.

Sample	Additional information	Ingredients *
A	Multiple kibbles, speciality recipe	Beef, soybean meal, soy flour, animal fat, brewers rice, soy protein concentrate, corn gluten meal, ground yellow corn, glycerin, poultry by-product meal, ground wheat, animal digest, pearled barley, calcium carbonate, calcium phosphate, salt, grilled sirloin steak flavor, dried green beans, dried potatoes, sulfur, Vitamin E supplement, choline chloride, zinc sulfate, ferrous sulfate, added color (Red 40, Blue 2, Yellow 5, Yellow 6), niacin, wheat flour, potassium chloride, L-Lysine monohydrochloride, vitamins, minerals, garlic oil, C-5900.
B	Mature dogs	Whole grain corn, chicken by-product meal, animal fat, soybean mill run, flaxseed, chicken liver flavor, lactic acid, corn gluten meal, potassium chloride, l-lysine, choline chloride, vitamin E supplement, iodized salt, vitamins, calcium carbonate, dicalcium phosphate, minerals, l-tryptophan, taurine, glucosamine hydrochloride, l-carnitine, chondroitin sulfate, phosphoric acid, beta-carotene, rosemary extract.
C	Multiple kibbles	Corn, soybean meal, beef & bone meal, ground wheat, animal fat (BHA used as preservative), wheat middlings, corn syrup, water sufficient for processing, animal digest (source of roasted flavor), propylene glycol, salt, apple, hydrochloric acid, potassium chloride, caramel color, vegetable blend (peas, carrots & green beans), sorbic acid, sodium carbonate, minerals, choline chloride, vitamins, calcium sulfate, titanium dioxide (color), red 40 lake, yellow 5, red 40, BHA, blue 2 lake, yellow 6 lake, blue 1, DL-methionine, yellow 6.
D	Small breed, aging care	Chicken meal, rice, brown rice, corn gluten meal, chicken fat, barley, natural chicken flavor, dried beet pulp (sugar removed), rice flour, dried egg product, anchovy oil, dried brewers yeast, potassium chloride, flaxseed, calcium carbonate, fructo-oligosaccharides (FOS), salt, choline chloride, sodium tripolyphosphate, DL-methionine, vitamins, taurine, salmon meal, trace minerals, glucosamine hydrochloride, tea (green tea extract), L-carnitine, chondroitin sulfate, marigold extract (*Tageteserectal*.), rosemary extract.
E	Affordable cost	Ground corn, wheat middlings, de-fatted rice bran, meat and bone meal, animal fat, salt, potassium chloride, animal digest, corn gluten meal, coline chloride, minerals, vitamins.
F	Easy to digest, grain free	Chicken, potatoes, chicken meal, pea protein, peas, sweet potatoes, poultry fat (preserved with mixed tocopherols), apples, pumpkin, natural flavor, tapioca starch, tomato pomace, salt, potassium chloride, choline chloride, vitamins, minerals, citric acid (used as a preservative), *Yucca schidigera extract*, rosemary extract.
G	Real salmon	Salmon, brewers rice, ground whole grain sorghum, potato, ground whole grain barley, chicken meal, fish meal, chicken fat, dried egg product, dried beet pulp, natural flavor, brewers dried yeast, potassium chloride, salt, sodium hexametaphosphate, calcium carbonate, dl-methionine, choline chloride, fructooligosaccharides, minerals, vitamins, beta-carotene, rosemary extract.
H	Low fat	Turkey, chicken, barley, brown rice, potato, rice, pea fiber, chicken meal, herring, natural flavors, chicken fat, flaxseed, apple, carrot, herring oil, sunflower oil, egg, cottage cheese, alfalfa sprouts, pumpkin, dried chicory root, L-carnitine, vitamins, minerals, direct fed microbials (dried *Lactobacillus acidophilus* fermentation product, dried *Lactobacillus casei* fermentation product, dried *Enterococcus faecium* fermentation product), lecithin, rosemary extract.

* Vitamin and mineral lists of all samples (except for sample B) are not all-inclusive.

The appearance attributes evaluated were brown color, color uniformity, shape uniformity, size uniformity, surface roughness, porous, oily, grainy, and fibrous. For aroma the panelists evaluated ashy, barnyard, broth, brown, cardboard, cooked, dusty/earthy, fish, grain, liver, meaty, musty/dusty, oily, oxidized oil, soy, smoky, spice brown, spice complex, stale, starchy, straw-like, toasted, and vitamin. For multiple kibble samples (A and C) all types of kibbles were combined for the evaluation, to mimic how the product is perceived by the pet owner.

### 2.3. Consumer Study

A Central Location Test (CLT) was conducted in the Sensory and Consumer Research Center at Kansas State University (Olathe, KS, USA). Participants (*n* = 100, men = 30, women = 70) were recruited from the Kansas City area. Participants were recruited from the Center’s database and were screened for age (>18 years old), dog ownership, not working in the pet food industry, and had to be personally responsible for at least 50% of dog food purchases. Among recruited pet owners, 66% had one dog in the household, 29% had two dogs, and 5% had 3 dogs. Participant demographics are shown in Table 2. 

**Table 2:** Demographics of the participants in the consumer study: gender, age, consumers annual income (US dollars), number of dogs owned.

Gender	Male						Female					
Consumers %	30%						70%					
**Age**	**18–24**	**25–34**			**35–44**		**45–54**		**55–64**			**>55**
Consumers %	2	25			22		35		12			4
**Income (USD)**	**<25,000**		**25,000–49,000**			**50,000–74,000**		**75,000–100,000**			**>100,000**	
Consumers %	3		14			26		33			24	
**Education level**	**Some high school**	**High school degree**			**Some college**		**College degree**		**Some graduate studies**			**Graduate degree**
Consumers %	1	3			14		44		10			27
**Number of dogs**	**One**			**Two**			**Three**			**Four or more**		
Consumers %	66			29			5			0		

For the evaluation, each pet owner used a tablet computer and computer questionnaires were administered by Compusense *at-hand* software (Compusense Inc., Guelph, ON, Canada). The number of participants for each session varied from 6 to 8. The samples were served monadically in a randomized order. Samples were presented in brown paper bags, lined with a plastic bag, containing approximately 900 grams of sample, a typical amount sold for purchase in small bags in the US. Bags were labeled with three-digit random codes. All eight samples were served to each participant during a 1-hour session. Participants were asked to open the bag and pour the sample into a ceramic pet bowl and then rate *overall liking, aroma liking*, *appearance liking, color, size, shape, uniformity,* and *oily appearance*. In addition, the participants were asked to indicate how much they thought their dog would like the sample using a 9-point hedonic scale where 1 = *dislike extremely* and 9 = *like extremely*. Pet owners were also asked about *aroma, oily appearance, uniformity shape, size*, and *color* intensities on a 5-point Just-About-Right (JAR) scale where 1 indicated “too weak”, 3 “just about right”, and 5 “too strong”. Moreover, participants scored their expected purchase intent (5 = *definitely would purchase*, 4 = *probably would purchase*, 3 = *may or may not purchase*, 2 = *probably would not purchase*, 1 = *definitely would not purchase*) and the expected cost of each sample (1 = *not at all expensive* to 5 = *very expensive*). Pet owners were also asked to describe their specific likes and dislikes for each sample using open-ended questions.

### 2.4. Data Analysis

Clusters among consumers were identified according to their *overall liking* score (Ward’s clustering procedure) using SAS^®^ statistical software (Version 9.3, SAS Institute Inc., Cary, NC, USA). Significant differences (*P* < 0.05) among products were determined using two-way analysis of variance and Fisher’s protected Least Significant Difference (LSD) in SAS^®^ statistical software (Version 9.3, SAS Institute Inc., Cary, NC, USA) for clusters among consumers according to their overall liking scores and Tukey’s HSD test in Compusense *at-hand* (Compusense Inc., Guelph, ON, Canada) for liking scores. Unscrambler software (The Unscrambler X version 10.2, Camo Software AS, Oslo, Norway) was also used to plot a liking map of samples fitted with consumers’ liking using Principal Components Analysis. Compusense *at-hand* (Compusense Inc., Guelph, ON, Canada) was used to collect consumer scores and analyze JAR data. For this data, scores 1–2 were grouped as “too low” and scores 4–5 were grouped as “too high”. Scores of 3 were considered as “just about right”. Correlation among overall liking, appearance, aroma, color, size, shape, uniformity, and oily appearance liking was calculated using Pearson correlation in Unscrambler *vs.* 2014.1.08 (Addinsoft, New York, NY, USA).

## 3. Results and Discussion

### 3.1. Descriptive Data

Descriptive sensory analysis showed a wide range of shapes, sizes, aromas, and flavors present in the samples. The aroma attributes common to all of the samples were barnyard and cooked (Table 3). Aroma attributes such as broth, grain, musty/dusty, straw-like, and vitamin, were detected in seven out of eight samples while brown and cardboard were present in six samples. Other attributes found were oxidized oil (B, D, G, H, A), toasted (C, D, E, F, A), meaty (C, D, F, A), soy (C, D, E, A), spice complex (C, D, E, F), stale (B, C, E, A), smoky (C, F, A), oily (C, E, F), fish (G, H), dusty/earthy (B, G), vegetable complex (C, A), burnt (D), liver (G), spice brown (F), starchy (D) and plastic (A).

Most of the scores were within the low intensity range (0–5 on a scale from 0 to 15), indicating the low overall aroma and the blended and complex nature of the product category, as was found by Di Donfrancesco *et al*. [9]. Sample D showed barnyard aroma notes in the moderate intensity range (5.5–10 on a scale from 0 to 15) with attributes such as cooked and oxidized oil scoring low-moderate and low for meaty and toasted notes. Descriptive analysis confirmed the specific characteristics expected to be present in the samples, such as the fishy notes in samples G and H, or the higher meaty notes in samples D and F. Sample D (the sample with no grain notes) had the highest levels of barnyard, broth, burnt, and cooked as well as meaty, musty/dusty, oxidized oil, soy, spice complex, starchy, straw-like, toasted, and vitamin within the sample set. Sample F presented the highest levels for brown and meaty, together with smoky and brown spice notes. Sample H had higher levels for both fish and oxidized oil, and lacked any presence of attributes such as broth or brown, present in most of the other samples. Plastic aroma was only present in sample A, probably due to processing or packaging. Sample C showed grain, smoky, oily, meaty, vegetable complex, and brown notes as well as the highest levels for stale aroma among the sample set. Oxidized oil aroma was not detected in this sample.

Samples G and F were characterized by a darker brown color while sample H was lighter (Table 3). Sample E showed the lowest shape and size uniformity within the sample set. Samples B, G, and E presented higher surface roughness than the other samples. Samples F and D showed low scores for both grainy and fibrous appearance.

**Table 3:** Aroma and appearance profiles from descriptive analysis (0–15 scale).

*Aroma profile from descriptive analysis (0–15 scale)*								
Sample #	A	B	C	D	E	F	G	H
Atribute								
Ashy	0	0	0	0	1.5	0	0	0
Barnyard	3.0	5.0	3.0	5.5	3.0	2.5	3.0	2.5
Broth	2.5	2.5	3.0	3.5	2.5	3.0	2.5	0.0
Brown	3.0	2.0	2.5	0.0	3.0	4.0	3.0	0.0
Burnt	0.0	0.0	0.0	3.0	0.0	0.0	0.0	0.0
Cardboard	3.0	4.0	2.5	0.0	3.0	0.0	3.0	3.0
Cooked	2.0	3.0	2.5	5.0	2.0	3.0	3.0	2.0
Dusty/Earthy	0.0	2.0	0.0	0.0	0.0	0.0	2.0	0.0
Fish	0.0	0.0	0.0	0.0	0.0	0.0	4.0	4.0
Grain	3.0	2.5	3.0	0.0	4.0	2.5	3.0	3.0
Liver	0.0	0.0	0.0	0.0	0.0	0.0	3.0	0.0
Meaty	3.5	0.0	3.0	4.0	0.0	4.0	0.0	0.0
Musty/Dusty	2.0	2.0	2.0	3.0	3.0	2.5	0.0	2.5
Oily	0.0	0.0	2.5	0.0	2.0	2.0	0.0	0.0
Oxodized Oil	2.5	2.5	0.0	5.0	0.0	0.0	3.5	3.0
Plastic	2.0	0.0	0.0	0.0	0.0	0.0	0.0	0.0
Soy	2.5	0.0	1.5	3.0	2.5	0.0	0.0	0.0
Smokey	2.0	0.0	2.0	0.0	0.0	2.0	0.0	0.0
Spice Brown	0.0	0.0	0.0	0.0	0.0	1.0	0.0	0.0
Spice Complex	0.0	0.0	2.5	3.0	1.5	1.0	0.0	0.0
Stale	2.5	2.0	2.5	0.0	2.0	0.0	0.0	0.0
Starchy	0.0	0.0	0.0	2.5	0.0	0.0	0.0	0.0
Straw-like	1.5	2.0	2	2.5	0.0	2.0	2.5	2.0
Toasted	1.5	0.0	1.5	4	2.5	2.0	0.0	0.0
VegetableCompl.	2	0.0	2	0.0	0.0	0.0	0.0	0.0
Vitamin	2.0	2.5	2.0	3.0	2.5	2.5	2.0	0.0
Brown color	Nd *	6.0	Nd *	7.0	7.0	12.0	12.0	3.5
Shape	Misc.	Nugget	Misc.	Round	Nugget	Square	Nugget	Nugget
Color Uniformity ** Uniformity ** Uniformity **	nd	99%	nd	70%	99%	98%	98%	98%
Shape Uniformity **	nd	93%	nd	60%	80%	95%	85%	95%
Size Uniformity **	nd	0.95%	nd	80%	80%	90%	90%	98%
Surface Roughness	nd	6.5	nd	2.0	6.0	2.0	5.0	3.0
Porous	nd	6.0	nd	2.0	4.0	2.0	6.0	3.5
Oily	nd	3.0	nd	5.0	2.0	3.0	2.5	2.0
Grainy	nd	4.0	nd	2.0	4.5	1.5	6.0	4.0
Fibrous	nd	2.0	nd	2.0	2.0	1.5	4.5	2.0

* Data relative to appearance of sample composed by multiple different kibbles (A and C ) are not shown. ** uniformity scores expressed as percentages.

### 3.2. Consumer Study Results

#### 3.2.1.Acceptability and Intensity Scores

Overall liking was significantly correlated with appearance, color, and aroma liking of the products. Sample C, one of the two samples composed of multiple kibbles, was the sample that was scored the highest overall liking score as well as highest scores for liking of appearance, shape, uniformity, oily appearance, and color (Table 4). The liking score for color in this sample represented the highest liking score observed in the study (average score >7).

**Table 4:** Consumers’ liking scores (1–9 hedonic scale).

Sample #	A	B	C	D	E	F	G	H
Atribute								
Overall liking	5.26bc **	5.70b	6.77a	5.15bc	5.79b	4.54c	4.83c	5.01bc
Appearance liking	5.24bcd	5.82bc	6.99a	5.24bcd	6.12ab	4.53d	4.86d	4.92cd
Aroma liking	5.00abc	4.69bc	5.56a	4.37c	5.27ab	4.63bc	4.69bc	4.97abc
Color liking	5.97bc	6.20bc	7.05a	6.29ab	6.32ab	5.02de	5.41cd	4.4e
Size liking	5.16bc	6.14a	6.32a	5.52ab	6.42a	4.14d	4.28cd	6.40a
Shape liking	4.98cd	6.55ab	6.84a	5.37cd	6.59ab	4.91d	5.80bc	6.43ab
Uniformity liking	5.02c	6.22ab	6.77a	5.77bc	6.25ab	5.50bc	5.69bc	6.08ab
Oily appearance liking	5.59ab	5.29b	6.08a	5.56ab	5.31b	5.26b	5.38b	5.48ab
Dog liking *	5.76b	5.99b	6.87a	5.85b	5.70b	5.28b	5.40b	5.30b

* Predicted by consumers; ** Different letter within a row indicates a significant difference among the samples (*P* < 0.05).

**Table 5:** Response to aroma, color, appearance, size, and uniformity intensity as too low, just about right (jar), or too high by % of consumers.

	AROMA			COLOR			OILY APPEARANCE			SIZE			UNIFORMITY		
Sample	Too low	JAR	Too high	Too dark	JAR	Too light	Too low	JAR	Too oily	Too small	JAR	Too large	Too low	JAR	Too high
A	19	48	33	31	58	11	26	73	1	16	46	38	54	44	2
B	9	43	48	6	72	22	11	61	28	6	56	38	5	67	28
C	6	61	33	14	79	7	12	76	12	5	67	28	20	77	3
D	5	34	61	21	72	7	8	65	27	43	53	4	7	69	24
E	11	50	39	2	70	28	34	64	2	24	71	5	8	70	22
F	11	37	52	60	39	1	9	68	23	75	25	0	6	68	26
G	13	43	44	46	52	2	18	68	14	76	24	0	9	65	26
H	33	49	18	0	26	74	36	63	1	27	68	5	2	76	22

**Figure 1:**
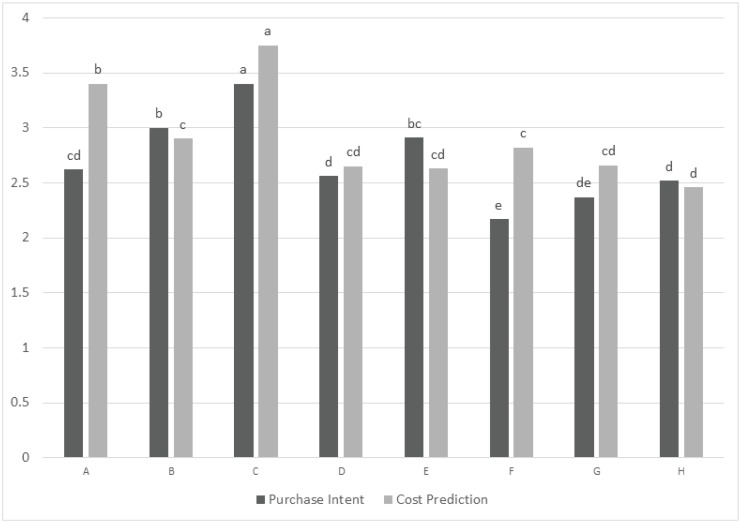
Purchase intent (1 = definitely would not purchase by consumers, 3 = may or may not purchase, 5 = definitely would purchase), predicted cost (1 = not at all expensive to 5 = very expensive) Different letters within a variable indicate a significant difference among the samples (*P* < 0.05).

Attributes such as aroma, color, oily appearance, and size also were scored for intensity (Table 5). Color intensity of kibbles in sample C was perceived by most of the consumers (79%) as ‘just about right’. In contrast to the other sample with different kibbles (Sample A) which was perceived as low in uniformity of shape by a large portion of consumers (54%), sample C was perceived as ‘just about right’ for shape uniformity by 77% of consumers. When asked to predict purchase intent and product price, consumers indicated this sample (C) was the one they were more willing to buy despite the fact that this sample also was perceived to be the most expensive product within the sample set (Figure 1). This could be explained by the positive correlation shown between perception of quality and price. Plassmann *et al.* [16] found that when a product was perceived as more expensive, the consumers rate it better than the product perceived as less expensive. Consequently, a low price perception is translated into a lower acceptability by consumers because of expected lower product quality. In this study, the highest predicted prices were earned by the two multi-shaped and multicolored kibbles products (C and A). However, the highest score (C) was 3.75 on a scale where 3 = *neither expensive nor inexpensive* and 5 = *very expensive*. Pet owners also predicted that sample C would be the most appreciated product by their dogs. This data suggest that consumers equate high price with high pet acceptability and appear willing to purchase more expensive dog foods. Except for sample C, the other samples in this study do not show a significant difference for consumers’ expectation of liking by their pet dogs and it is not possible to verify if consumers relate low predicted price with low pet acceptability.

Despite the actual low cost on the market, sample (E), characterized by a nugget shape and a moderate brown color, was the second highest in overall liking and appearance liking. However, it scored almost one full point lower than sample C for both of the attributes (Table 4). Sample E also had the highest score for size liking and the second highest score for aroma, shape and uniformity liking (average scores >6).

The least liked product was sample F, which was dark brown and square-shaped and with the highest oily appearance among samples, based on the descriptive analysis. It showed the lowest liking scores for size, shape, and oily appearance. This sample earned the lowest predicted ‘dog liking’ score, but was only slightly lower than other samples such as G (nugget-shaped and dark brown) or H (nugget-shaped and light brown). The sample perceived as having the lowest cost was the sample that was scored the lowest for color liking (sample H).

Sample F, one of the most expensive products in the market, was not perceived as the most expensive by consumers. This product was grain free, included natural ingredients and was claimed to favor an easy digestion. Sample F received the lowest scores for appearance liking, size liking, shape liking, and oily appearance liking as well as for overall liking. The low-cost sample (E) was not perceived as the lowest cost sample by consumers (Figure 1). Sample E had a remarkably lower cost than other samples in the market, and earned some of the highest scores for attributes such as aroma liking and appearance liking. The low nutritional value and the low protein quality was probably counterbalanced in consumer’s perception by the appearance (not too dark and uniform brown color, high surface roughness, moderate porousness, fibrousness, and low oily appearance) and a high level of grain aroma notes with absence of oxidized oil aroma.

When asked to comment on their likes and dislikes for the products, the consumers focused on overall appearance of samples. For instance, consumers’ comments indicated that sample C was appreciated mostly because of the variety of colors, shapes, sizes, and textures. To some consumers this was associated with a variety in ingredients and flavors, while others perceived it as an indicator of healthiness or freshness of the product. For sample F, the least liked sample, most of the comments pointed out that the kibbles were too small, it was too dark in color, and had a poor smell. The o-shaped sample (D) was one of the least liked among the set, and earned comments about its similarity with a well-known breakfast cereal product. For some consumers this was a good aspect, while some others considered this shape odd for a dog food product and more appropriate for cat food. Some consumer even considered this shape dangerous because of the possibility for a child to mistake the product for a breakfast cereal. Some consumers appreciated sample A for its variety of colors and sizes. However, other consumers highlighted the low uniformity of the sample together with kibble shapes that was considered strange, especially one kibble that was irregularly shaped and seemed like an imitation of a chunk of meat.

Average aroma liking scores fell in a range between ‘*dislike slightly*’ and ‘*like slightly*’. Samples E and C were the only samples scored higher than 5 for aroma liking on the average. Although descriptive sensory data showed some unique aroma characteristics that distinguished these samples (e.g., a higher level of grain aroma (4.0) for sample E and vegetable complex notes for sample C) it’s possible that the particular combinations of aroma attributes present in these samples, more than a single note, originated the highest aroma liking scores by consumers. In addition to the notes present in their aroma profile, it has to be highlighted that both of the samples did not show any oxidized oil notes. Consumers’ comments also indicated that sample C was appreciated because of a mild aroma. Although sample C was not the only sample showing a total absence of oxidized oil aroma, this sample also exhibited a lack of many other attributes such as ashy, dusty/earthy, fish, liver, plastic, spice brown, and starchy. This likely is related to the mild aroma perception noted in the consumers’ comments.

#### 3.2.2. Consumer Clusters

Cluster analysis of consumers based on overall liking scores showed six clusters among consumers who participated in the study (Table 6). These clusters, based on individual consumer variation in liking scores showed little or no relation to income, age, gender, or education.

**Table 6:** Consumer clusters according to overall liking.

Sample#	Cluster 1 (n = 19)	Cluster 2 (n = 17)	Cluster 3 (n = 15)	Cluster 4 (n = 21)	Cluster 5 (n = 10)	Cluster 6 (n = 18)
A	3.7 d *	7.8 a	4.8 c	6.0 b	7.3 a	2.8 d
B	6.8 a	5.6 bc	3.6 d	6.3 ab	6.6 ab	5.2 c
C	7.7 ab	7.8 ab	7.0 bc	6.3 c	8.1 a	4.4 d
D	6.2 a	4.0 c	5.9 ab	6.5 a	4.5 bc	3.3 c
E	6.2 ab	5.3 bc	4.1 d	6.9 a	4.5 cd	6.7 a
F	3.7 cd	3.2 d	5.3 b	6.6 a	3.0 d	4.6 bc
G	3.8 b	4.6 b	4.7 b	6.5 a	1.4 c	6.2 a
H	5.8 a	5.5 ab	4.3 b	5.8 a	2.5 c	4.8 ab

* Different letter within a row indicates a significant difference among the clusters (*P* < 0.05).

Cluster 1 included consumers who did not like the two samples perceived as too small (F and G) and the multiple kibble product with low shape uniformity (sample A). On the contrary this cluster provided one of the highest scores for the other multiple kibble sample (C). Consumers in cluster 2 gave scores below 6 for all of the samples except the two with multiple kibbles. Cluster 3 showed scores below 6 for all of the products except for sample C. On the contrary, cluster 4 (n = 21), the largest cluster, liked all of the products but all of the scores were within the 5.8–6.9 range. The 5.8 average score for sample H was higher than the average score it earned for overall liking by all of the participants (5.0). Cluster 5 (n = 10) seemed to be positively influenced by appearance variety and large dimensions with consumers providing the highest scores to multiple kibbles and products perceived as large in size. The average score for sample C, the most liked overall by consumers in this cluster, was 8.1 (overall data average was 6.8). These consumers also showed the lowest scores for the products perceived as too small (sample F and sample G). Cluster 6 (n = 18) was formed by consumers that provided the lowest scores for the multiple kibble products, samples C and A (multi-colored and multi-shaped), compared to the scores these sample earned in the other clusters. It is possible to consider this consumer cluster as negatively influenced by appearance variety and large kibble dimensions. However, products perceived as too small (consumer intensity score) did not earn the highest score by consumers of this cluster either.

**Figure 2:**
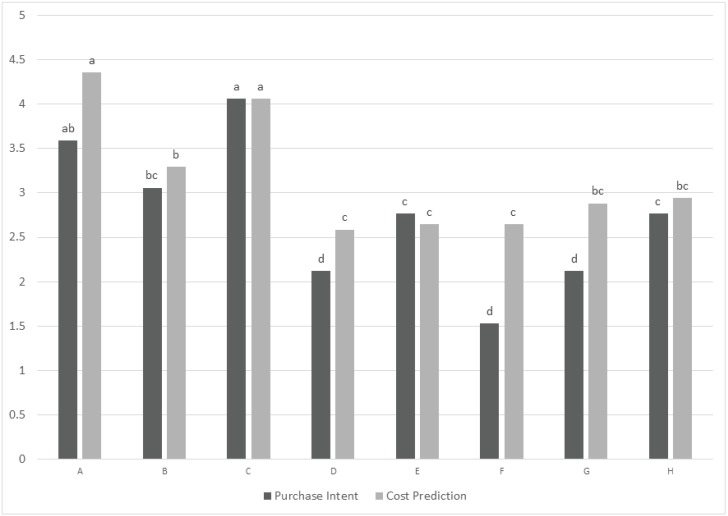
Cluster 2: purchase intent (1 = definitely would not purchase by consumers, 3 = may or may not purchase, 5 = definitely would purchase), predicted cost (1 = not at all expensive to 5 = very expensive). Different letters within a variable indicate a significant difference among the samples (*P* < 0.05).

**Figure 3:**
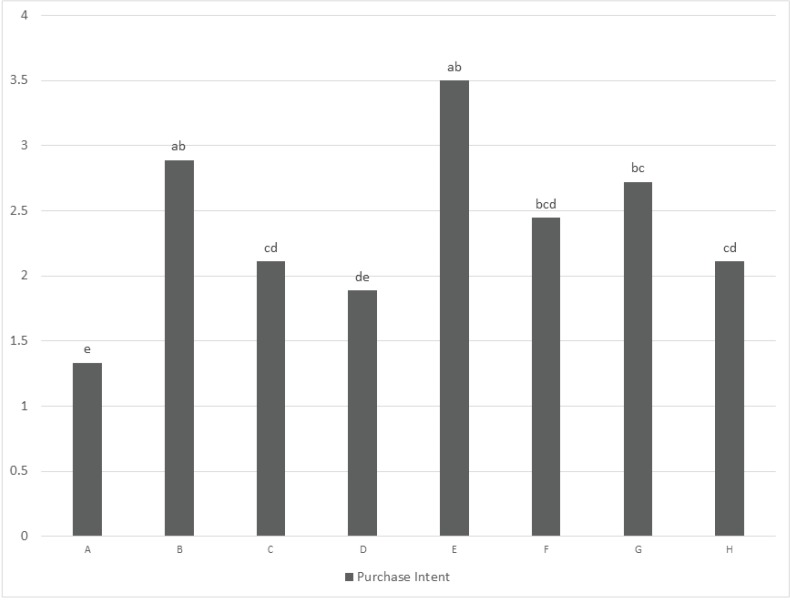
Cluster 6: purchase intent (1 = definitely would not purchase by consumers, 3 = may or may not purchase, 5 = definitely would purchase). Different letters within a variable indicate a significant difference among the samples (*P* < 0.05).

Purchase intent and cost prediction within each of the six clusters also was analyzed confirming that for most of the clusters, purchase intent and cost prediction were linked with overall liking. For example in cluster 2 (Figure 2) both purchase intent and cost prediction followed the overall liking scores of the cluster, with sample C and A earning the highest scores and sample D, F, and G the lowest for purchase intent. In Cluster 6 (Figure 3), although there was not a significant difference related to cost prediction among samples, samples were significantly different for purchase intent and showed a trend similar to the overall liking scores of the cluster. This cluster was the only one where sample C was not preferred over the other samples and, thus, it was possible to observe an potential relationship to purchase intent. The other multiple kibble product (sample A) that earned the lowest overall liking score in this cluster, also earned the lowest purchase intent score.

Consumers were asked to indicate the brands that they usually purchased and this information was related to the different clusters. Clearly current consumer purchase behavior can influence consumer acceptance scores. Approximately 50% of consumers in cluster 5 usually purchased the Kibbles ‘n Bits brand, a multi-colored, multi-shaped kibble product. The consumers in this cluster provided the highest overall liking score (8.1) to sample C (a multi-colored and multi-shaped product). Finally cluster 6 was characterized by 44% of consumers purchasing Science Diet. Conversely cluster 6, which contained a high proportion (44%) of Science Diet users (a highly uniform product), scored products with low uniformity (A, C) low in overall liking. No other cluster contained such high percentages of brand users. The most purchased brands by consumers in cluster 1 were Iams (21.1%), Purina One (26.3%), Science Diet (31.6%). In cluster 2 there was no brand purchased by more than 20% of consumers with the most purchased brands represented by Science Diet and Purina One (17.6%), Iams, Purina Proplan, and Kibble ‘n Bits (11.8%). In cluster 3 the most purchased brands were Purina One (26.7%), Purina Proplan and Purina Beneful (20%). In cluster 4, brands like Science Diet, Purina One (19%), Iams, and Kibble ‘n Bits (14.3%) were still the most purchased. 

#### 3.2.3. Drivers of Liking and Disliking

The products used were commercially available products and did not attempt to hold specific variables constant. Thus, the aspects that appear to drive liking or disliking are based on overlapping characteristics and with only eight products represent a general overview. Specific aspects would need to be studied in more controlled studies or with larger numbers of products to determine specific relationships. 

**Figure 4:**
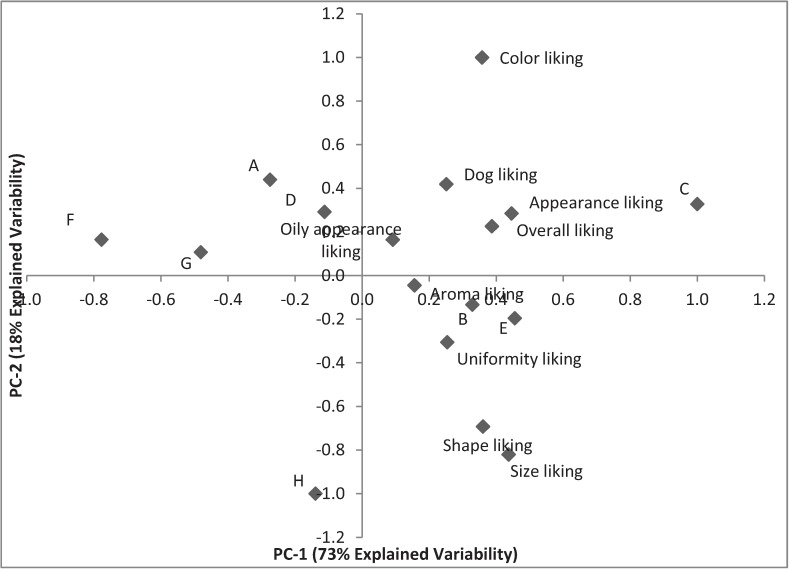
Principal Components Analysis based on consumer liking.

Generally, overall liking was greatly influenced by the appearance of the samples (Figure 4). Among the different specific appearance characteristics, color liking was highly related to the overall liking scores by dog owners. Overall, the most liked sample was a multiple-colored and -shaped kibble product and the two least liked samples were the two darkest brown samples according to the descriptive analysis. It is also possible to notice that prediction of dog liking was highly correlated with the consumers’ overall liking (r = 0.928).

**Table 7:** Correlation between consumers’ overall liking and the other liking scores.

Correlation *r*	Aroma liking	Appearance liking	Color liking	Size liking	Shape liking	Uniformity liking	Oily appearance liking	Dog liking
Overall liking	0.762	0.993	0.809	0.705	0.721	0.754	0.697	0.928
*p*-value	0.028	<0.0001	0.015	0.051	0.044	0.031	0.055	0.001

Aroma did not drive consumer’s overall liking and does not appear to drive acceptance as much as appearance, as shown in the correlation analysis (Table 7). However, aroma may have some impact on individual consumer clusters. For example, consumers in cluster 5 gave the lowest scores (<3 point-scale) to the only two sample showing fish aroma notes from descriptive analysis (G and H). Another example could be sample G that was perceived as too small by owners but also was the only sample that showed liver aroma notes. The aroma component may have contributed to the extremely low score (1.4) earned from owners in cluster 5 with all the other clusters scoring in a 3.8–6.5 range.

Expected cost did not seem to drive overall acceptance as one of the two products that scored highest for expected cost was the fourth most liked sample.

### 3.3. Discussion

Descriptive analysis showed the tested samples were different from each other and offered a wide range of characteristics to study across the samples. This enabled analysis of these different characteristics with the aim of understanding the aspects that drive consumer’s acceptance of dry dog food when brand and packaging are removed. Clearly, appearance is a key driver for acceptance of dry dog food, a product that is not eaten by the actual purchaser. Other examples of aspects that were important for consumer segments were the overall intensity perception of aroma or the presence of particular aroma notes.

Some similarities among products helped clarify if a characteristic influenced consumer liking. An example of this was the influence of appearance and not aroma on overall liking for the sample composed of multiple different colored and shaped kibbles (C). It was also characterized by aroma attributes such as smoky, broth, grain, meaty, toasted, and musty/dusty. Sample C was the most liked among consumers while sample F, with similar aroma characteristics, was the least liked overall within the sample set. This indicates the importance of visual stimulation in overall liking of dry dog foods. When asked about color intensity, sample F was perceived by 60% of consumers as *too dark*. Specific characteristics of samples also highlighted the lower influence of aroma on pet owners liking scores. For instance, the sample with the highest scores for oxidized oil aroma based on descriptive analysis (sample D) showed the lowest score for aroma liking, but was not the least liked overall.

The results indicated that multiple kibbles in a product are not enough to drive consumer’s liking, and that an optimal combination in color, shape, size, and oily appearance has to be pursued. Although sample C was highly liked, the other sample with multiple kibbles (A) was one of the least liked by consumers, and was scored the lowest in uniformity liking among the samples. Sample A contained a particular kind of kibble, characterized in descriptive analysis by its large size, highly irregular shape, and high springiness, which was different from sample C.

Pet owners were not accurately able to predict how expensive/inexpensive the dog food was based on appearance and aroma alone. Multiple kibbles were related to cost expectation; the samples composed of multiple kibbles were perceived as the most expensive samples in the set. Sample H was perceived as the least expensive product and it also was the sample earning the lowest color liking scores. These results show that cost prediction is related with color liking but at the same time the cost prediction is not driving the overall liking.

Previous studies have shown that appearance plays a major role, among sensory characteristics, in determining consumers purchase intent [17]. Moreover, for new products and products that consumers have not previously tasted, appearance is even more important in driving consumer preference [18]. Consumers’ intensity and liking scores indicated that a too small kibble size may not be appreciated by pet owners. Samples G and F were perceived as too small by consumers (respectively 76% and 75%) and consequently earned the lowest scores for size liking. It was observed from clustering analysis that different consumer clusters like different sized kibbles. This aspect may be related to dog size and breed and future studies can help clarify that relationship.

Based on intensity scores given by consumers, it is possible to observe that consumers did not appreciate a strong aroma in their dog food. Sample D had moderate aroma intensity scores from descriptive analysis, compared to low aroma intensity scores of the other samples and was the only sample not scored for grainy attribute in its aroma profile. This sample was perceived as having too strong aroma intensity by 61% of consumers and earned the lowest score for aroma liking but was only the fourth least liked overall confirming that aroma characteristics did not drive the overall liking as the appearance characteristics did. The information related to the grainy aroma attribute can indicate a primary role of grainy aroma in the aroma liking of dry dog food. When grainy aroma was absent, the aroma liking scores were low. Furthermore, when the grainy aroma was stronger, like was noted with the low-cost product, the aroma liking score was higher as well.

Previous studies have looked at the human-dog relationship and the epidemiological data that can be related to dog obesity [19,20,21]. According to Suarez *et al.* purchase behavior of commercial dog food is different for owners of normal weight dogs when compared to the owners of overweight dogs [19]. These authors found that owners of overweight dogs are more interested in special prices and promotions and less interested in the correct dog nutrition than were the owners of normal weight dogs. Similar findings regarding the price of dog food were reported by Kienzle *et al.* [20]. These authors also found that the owners of overweight dogs tended to overhumanize their pet, thus, some of the needs of their pets, such as exercise, were ignored. This was confirmed by Courcier *et al.* [21] who found a correlation among dog obesity and exercise hours per week. Furthermore, they found that feeding snacks and treats to the dog is a risk factor for being overweight. Pet obesity is a serious issue and sensory properties of dog food as perceived by the owner and as perceived by the pet have not been studied in relation to being overweight. Future studies should address the possible associations among aroma, flavor, and texture and quantities served and consumed, further.

## 4. Conclusions

Eight dry dog food samples, with different appearance and aroma characteristics, were evaluated by a descriptive panel and by pet owners. The study indicated that consumers’ liking was most influenced by the appearance of the products. Product color intensity, especially darker brown colors, seems to have a negative relation with pet owners’ liking. In addition, kibble size also influenced product liking, with products where kibbles are perceived as too small showed the lowest overall liking scores. Cluster analysis showed a group of consumers who liked large kibble size more than other participants, but the same was not seen for small kibbles. Uniformity of shape within a product can also play an important role in dry dog food acceptance. Of the two multi-colored and multi-shaped kibbles products, one resulted the most liked and the other, perceived as too low in uniformity of shape (particularly related to one of its kibbles), earned a score almost 2 points lower for appearance liking. The brands consumers usually purchased seem to be associated with overall liking at least for some consumer segments.

Aroma of dry dog food seems to have less of a role in driving consumers liking and their willingness to purchase specific dry dog foods than appearance. However, for products where the aroma was perceived as too strong, was disliked, or had higher the presence of off-flavor notes (e.g., oxidized oil, musty/dusty), overall liking was impacted negatively. This indicates that pet owners are generally accepting of “dog food aroma” or specific flavor notes associated with the product.

Interestingly, the presence of grain-type aroma notes seemed to increase liking by consumers and a lack of that note seemed to penalize the product. This may be related to the modulation of aroma by that relatively bland aroma characteristic.

Based on this research, dry dog food products will be well liked by pet owners if the kibbles vary in color and shape with high uniformity within a single kibble type, and a low aroma that includes grain-type notes. Including different colors and shapes of kibbles also can increase the perception of a more expensive product.
